# Sickle cell disease: understanding pathophysiology, clinical features and advances in gene therapy approaches

**DOI:** 10.3389/fphar.2025.1630994

**Published:** 2025-08-22

**Authors:** Muhammad Taher, Sofea ‘Aisyah Aminondin, Nur Asyilah Nasir, Noor Afiqah Jasmadi, Nur Irdeena Nabella Nizam, Ilhan Syahmi Shahrul, Deny Susanti, Junaidi Khotib, Md Faiyazuddin, Riyanto Teguh Widodo, Muhammad Salahuddin Haris

**Affiliations:** ^1^ Faculty of Pharmacy, International Islamic University Malaysia, Kuantan, Malaysia; ^2^ Department of Chemistry, Faculty of Science, International Islamic University Malaysia, Kuantan, Malaysia; ^3^ Department of Pharmacy Practice, Faculty of Pharmacy, Airlangga University, Surabaya, Indonesia; ^4^ Centre for Global Health Research, Saveetha Institute of Medical and Technical Sciences, Chennai, India; ^5^ Department of Pharmaceutical Technology, Faculty of Pharmacy, Universiti Malaya, Kuala Lumpur, Malaysia; ^6^ Department of Pharmacy, Faculty of Pharmacy and Health Sciences, Royal College of Medicine Perak, Universiti Kuala Lumpur, Ipoh, Malaysia

**Keywords:** anemia, gene therapy, gene editing, CRISPR, hemoglobinopathies, hematopoietic stem cell transplantation

## Abstract

Sickle cell disease (SCD) is an inherited blood disorder marked by the production of abnormal hemoglobin, leading to the distortion—or sickling—of red blood cells. The SCD arises from a single-point mutation that substitutes glutamic acid with valine at the sixth codon of the β-globin chain in hemoglobin. This substitution promotes deoxyhemoglobin aggregation, elevating red blood cell stiffness, and triggering vaso-occlusive and hemolytic repercussions. To explore therapeutic advances in tackling this disease, this review analyzed articles published from January 2015 to January 2025 using the three databases using relevant keywords focusing on SCD and advancement in therapy. It was found that allogeneic hematopoietic stem cell (HSC) transplantation can alleviate symptoms but is limited by a shortage of well-matched donors and immunological challenges. In contrast, autologous gene-modified HSC transplantation via gene therapy offers comparable therapeutic benefits without associated immunological complications. Clinical trials utilizing lentiviral vector-mediated gene insertion have demonstrated promising therapeutic outcomes by preventing hemoglobin aggregation. Emerging gene editing approaches such as CRISPR/Cas9 are expanding treatment options, marking the transition of SCD gene therapy from theoretical concept to clinical application.

## 1 Introduction

Normal red blood cells are disc-shaped and flexible cells that transport oxygen bound to hemoglobin throughout blood circulatory system. Sickle cell disease (SCD) is an autosomal-recessive genetic condition. Sickle cell hemoglobin occurs when the red blood cell becomes sickle-shaped, giving the disease its name. An individual became a sickle cell trait when they received a single gene mutation of the inherited disease. A person will acquire the disease when they attain two faulty hemoglobin genes, with both hemoglobin S gene inherited from each parent, or inherit one hemoglobin S from one parent, and another faulty hemoglobin gene, such as beta (β) thalassemia, hemoglobin C, hemoglobin D, or hemoglobin E, from the second parent (NHLBI, 2024; Johns Hopkins Medicine, 2024). The likelihood of a child being born with SCD increases significantly if both parents are carriers, with a 25% probability of inheritance per pregnancy.

**FIGURE 1:**
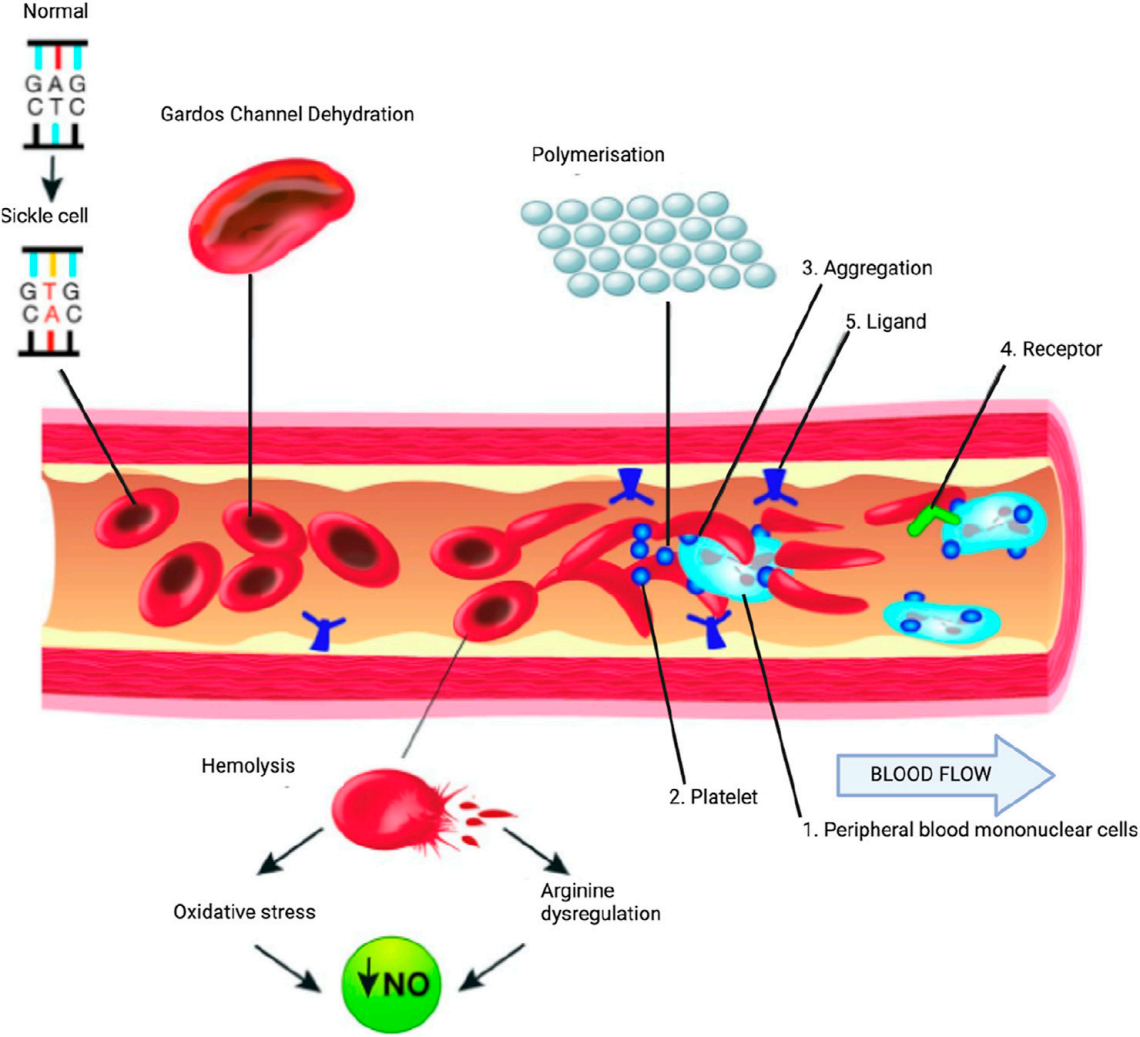
Partial pathophysiological model of sickle cell anemia. A single gene mutation (GAG→GTG and CTC→CAC) results in a defective hemoglobin that when exposed to deoxygenation (depicted in the right half of the diagram) polymerizes (upper right of the diagram), resulting in the formation of sickle cells. Vaso-occlusion can then occur. The disorder is also characterized by abnormal adhesive properties of sickle cells; peripheral blood mononuclear cells (1) and platelets (2) adhere to the sickled erythrocytes. This aggregate is labelled (3). The mononuclear cells have receptors (4) (e.g., CD44 that bind to ligands (5), such as P-selectin that are upregulated. The sickle erythrocytes can also adhere directly to the endothelium. Abnormal movement or rolling and slowing of cells in the blood also can occur. These changes result in endothelial damage. The sickled red cells also become dehydrated as a result of abnormalities in the Gardos channel. Hemolysis contributes to oxidative stress and dysregulation of arginine metabolism, both of which lead to a decrease in nitric oxide (NO) that, in turn, contributes to the vasculopathy that characterizes sickle cell disease. Taken and modified from Gardner (2018) under http://creativecommons.org/licenses/by-nc/4.0/ policy.

A simplified overview of the pathophysiological mechanisms in SCD is illustrated in Figure 1. Upon deoxygenation, the sickle hemoglobin becomes insoluble and polymerizes into stiff fibers, leading to red blood cell sickling (Gardner, 2018). Due to their rigid shape, the cells are prone to occlusion in the microvasculature, causing downstream tissues to experience reduced blood flow, oxygen deprivation, and resulting ischemic injury or cell death. The impaired perfusion may subsequently lead to tissue necrosis or reperfusion injury (Gardner, 2018).

There are several variants of SCD including hemoglobin SS (HbSS), HbSC, and HbS/β-thalassemia. HbSS is the most common and severe form of disease. It occurs when an individual inherits one sickle cell gene from each parent. HbSC and HbS/β-thalassemia are milder types of SCD. In HbS/β-thalassemia, the individual inherits one HbS gene and one β-thalassemia gene, resulting in impaired hemoglobin synthesis and systemic disruption of oxygen transport. β-thalassemia exists in two forms: β^0^ (complete absence of β-globin) and β^+^ (partial reduction in β-globin production). Individuals with HbS/β^+^-thalassemia usually exhibit a milder phenotype, while HbS/β^0^-thalassemia is often associated with more severe symptoms (John Hopkins Medicine, 2024; Mayoclinic, n.d.).

It is estimated that approximately 100,000 individuals in the United States and several million worldwide are affected by SCD. The disease predominantly affects individuals of African, Middle Eastern, Indian, and Mediterranean descent. The presence of HbS among Malay individuals may reflect genetic admixture from diverse ancestral lineages, including South Asian and African populations (Inusa et al., 2019; Deng et al., 2015).

In a healthy individual, hemoglobin consists of two α-globin and two β-globin chains, forming a tetramer responsible for oxygen transport. Sickle hemoglobin results from a single nucleotide mutation in the β-globin gene (Hbβ) (Inusa et al., 2019). Specifically, this mutation replaces adenine with thymine at codon 6 of the β-globin gene. The substitution leads to the replacement of negatively charged glutamic acid with hydrophobic valine. The valine-type hemoglobin produces sticky patches on the protein surface that causes Hbs polymerizes into fiber and causing red blood cells to sickle and stiffen under low oxygen conditions. These sickled cells can obstruct blood flow, resulting in ischemia, tissue infarction, and hemolysis. These events trigger acute, severe pain episodes known as sickle cell crises. While normal have a lifespan of around 120 days, sickled RBCs typically survive only 10–20 days due to accelerated hemolysis (Sickle Cell Disease - Causes and Risk Factors | NHLBI, 2024; Inusa et al., 2019).

## 2 Methodology

This review was conducted by examining English-language articles published between January 2015 and August 2024 that explored the relationship between gene therapy and sickle cell disease, including associated clinical outcomes. The primary source of literature retrieval was the Scopus, PubMed and Google Scholar databases. Articles were identified using a combination of relevant keywords, including (“gene therapy” OR “gene editing” OR “genetic modification” OR “genetic engineering”) AND (“sickle cell” OR “sickle cell disease” OR “sickle cell anemia” OR “hemoglobinopathy”) AND (“advances” OR “developments” OR “innovations” OR “progress”) AND (“treatment” OR “therapy” OR “intervention” OR “management”) AND (“clinical trial” OR “study” OR “research” OR “evaluation”) (www.scopus.com).

Out of approximately 100 relevant publications and reviews, 65 were selected based on their relevance and alignment with the scope of this study. Articles were selected according to predefined inclusion and exclusion criteria to ensure appropriate data extraction and analysis. Thematic patterns within the selected studies were identified and analyzed using content analysis and principles of grounded theory.

## 3 Clinical features

Clinical features of SCD typically emerge in the latter half of the first year of life, as the predominance of fetal hemoglobin (HbF) declines and is replaced by adult hemoglobin (Inusa et al., 2019). The symptoms of SCD may resemble those of other hematological or systemic conditions, posing diagnostic challenges in early life. Common clinical presentations in patients with SCD include vaso-occlusive crises (pain episodes), chronic hemolytic anemia, acute aplastic crises, increased susceptibility to infections, and splenic sequestration episodes (Inusa et al., 2019).

### 3.1 SCD complications

The aforementioned issues are emphasized as those that impact infants and young children because they become immediately important following newborn screening. Young adults, teenagers, and older children suffer a variety of chronic complications.

#### 3.1.1 Stroke

The main blood arteries that provide oxygen to the brain may get blocked by the malformed cells. Severe brain injury can occur from any disruption in the blood and oxygen supply to the brain. The risk of experiencing a second or third stroke is increased if patients have sickle cell anemia.

#### 3.1.2 Jaundice

Due to their shortened lifespan, sickled red blood cells undergo rapid hemolysis, overwhelming the liver’s capacity to clear them. The breakdown of hemoglobin releases bilirubin, which accumulates in the body and causes jaundice (Idris et al., 2022). Elevated bilirubin levels may precipitate gallstones (cholelithiasis) and lead to cholecystitis.

#### 3.1.3 Priapism

Priapism is a prolonged, often painful erection that may occur with or without sexual stimulation. It results from obstruction of penile blood flow by sickled cells and may cause erectile dysfunction if treatment is delayed. Ischemic priapism is the most prevalent manifestation in children and adults with SCD (95%), with around 33% of adolescents and adults with SCD reporting experiencing it at least once (Idris et al., 2022).

Blood and other tests could be performed in addition to a thorough medical history and physical examination. Babies are screened for sickle cell disease, to reduce the chance of problems with early identification and treatment. Hemoglobin electrophoresis is a blood test that can identify either the patient is having sickle cell disease or is a carrier of the sickle cell gene, to confirm the diagnosis of sickling. A sample’s percentage of each hemoglobin type can be found via this method (Ashorobi et al., 2024). Patients with sickle cell trait have a combination of normal hemoglobin A and hemoglobin S (Ashorobi et al., 2024). Genetic testing can also be done on a pregnant mother.

In the process of determining the treatment, doctors need to take into account patients' health, age and other factors. Most people with sickle cell anemia do not have a cure (Mayoclinic, n.d.). The treatments indicated can alleviate pain and aid in avoiding the disease’s complications promptly.

## 4 Treatments

Treatment for sickle cell disease can be aided by early identification and prophylaxis of complications. The goals of treatment are to treat the symptoms, avoid infection, and protect vital organs from harm like stroke (Dampier et al., 2023). Treatment options could be.

### 4.1 Pain medications

The various approaches to pain management in sickle cell disease (SCD) vary depending on whether the patient is opioid-tolerant or opioid-naïve, and whether the pain is acute, chronic, or a combination of the two (Okpala and Tawil, 2002). Analgesics such as paracetamol and ibuprofen are generally given promptly to treat mild pain, while morphine is indicated for moderate pain (Clinical Practice Guidelines, n.d.; Johns Hopkins Medicine, 2024).

### 4.2 Blood transfusions

During a red blood cell transfusion, a sickle cell anemia patient receives red blood cells intravenously from a supply of donated blood. This lessens symptoms and complications by raising the quantity of healthy red blood cells. These are used to treat and prevent complications, such as stroke, in SCD.

### 4.3 Vaccinations and antibiotics

Penicillin is a general treatment for infection in SCD. Children who are susceptible to infections due to low immunity receive them as early as 2 months old to 5 years old. Adults with SCD might need to take penicillin for lifetime especially when they have pneumonia or splenectomy (Mayoclinic, n.d.). Children should receive all recommended vaccinations to prevent further disease, particularly for children with SCD due to their low immunity. In virus outbreaks, patients with SCD should take extra precautions and stay isolated.

### 4.4 Hydroxyurea

This medication helps reduce the frequency of pain crises, episodic acute chest syndrome, blood transfusions and duration of hospitalization, and slow down damages done to organs. Hydroxyurea increases the fetal hemoglobin (hemoglobin F). It works by keeping the red blood cell shape bigger, rounder, more flexible, and reduces the likelihood of turning sickle. The majority of sickle cell disease patients who take hydroxyurea possess few or no side effects. The majority of side effects are mild (Agrawal et al., 2014).

### 4.5 Bone marrow transplant

Healthy bone marrow from a donor is used to replace bone marrow that has been affected by sickle cell anemia. A matched donor without sickle cell anemia, such as a sibling, is typically used in the procedure. A bone marrow transplant is only advised for patients—typically children—who have severe sickle cell anemia symptoms and complications due to the risks involved, which include death (Ashorobi et al., 2024). The sole recognized treatment for sickle cell anemia is a stem cell transplant. Gene therapies and adult stem cell transplantation are currently undergoing clinical trials (Mayoclinic, n.d.).

Patients with sickle cell trait have a good prognosis, despite the fact that the trait has been linked to numerous complications, including exercise-induced death, splenic infarction, asymptomatic bacteriuria, and papillary necrosis (Ashorobi et al., 2024). The average life expectancy of individuals with sickle cell trait is equal to that of the general population, notwithstanding the complications that come with having the trait (Tsaras et al., 2009).

## 5 Standard care for SCD

Standards of care for sickle cell disease (SCD) include comprehensive management at different referral levels of healthcare, with recommendations tailored to each level (Paintsil et al., 2023). Supportive care has been the primary management approach. In addition to hydroxyurea, other new FDA-approved disease modifying agents namely, L-glutamine, crizanlizumab, and voxelator are also available since 2017, providing another option in medical settings (Higgins et al., 2022; Migotsky et al., 2022). The management of SCD major complications such as acute chest syndrome, pain and stroke involves the use of hydroxyurea and transfusion therapy (Howard, 2016). The National Heart, Lung, and Blood Institute guidelines provide evidence-based recommendations for the proper management of SCD, including health maintenance and the use of hydroxyurea (Evidence-Based Management of Sickle Cell Disease: Expert Panel Report, 2014 | NHLBI, 2014).

Not just limited to disease-modifying agents, standard care for SCD also includes transfusion programs, and early detection of organ failures. A high level of care can reduce the incidence of end-stage organ disease, but solid organ transplantation is a recognized treatment for patients who do develop the disease (Sharpe et al., 2023), though it is not widely accessible. The optimal approach to clinical care in pediatric patients has significantly improved through the advancement of screening and early detection techniques, along with the increased utilization of red cell transfusion and hydroxyurea (Hoppe and Neumayr, 2019). Moreover, gene therapy and hematopoietic stem cell transplantation present promising prospects in adults with chronic organ damage as potential therapeutic options (National Academies of Sciences, Engineering, and Medicine et al., 2022).

On top of that, the gene therapy approach must be relevant and equitable, depending on the reduction of health disparities and the distribution of outcomes across subgroups (Goshua et al., 2022). Implementing standard care based on evidence-based practices can improve the treatment of veno-occlusive episodes (VOEs) and the quality of care for SCD patients. Overall, a holistic approach to care, early intervention of complications, and the use of disease-modifying agents are important components of standard care for sickle cell disease (Kim et al., 2017).

## 6 Comparison between SCD interventions

Disease-modifying agents, such as hydroxycarbamide and long-term red blood cell transfusions, are commonly used in SCD to prevent complications and improve outcomes. The main purpose of hydroxycarbamide is to avoid recurring veno-occlusive episodes; patient compliance and dosage determine the medication’s effectiveness and adverse effects (Pondarré and Lionnet, 2023; Azmet et al., 2020). Besides hydroxycarbamide, long-term transfusion is another alternative either as first-line or second-line treatment for prevention of recurrent vaso-occlusive events (Al-Riyami and Daar, 2018).

Furthermore, early intervention in end-organ complications is crucial in SCD, as it can lead to better treatment outcomes and improved overall prognosis. Significant morbidity and death are associated with pulmonary complications in people with sickle cell disease (SCD), including acute chest syndrome and pulmonary hypertension. Another prominent and devastating side effect of sickle cell disease (SCD) is cerebrovascular illness, which includes stroke and silent brain infarct (Elendu et al., 2023). Limited data indicate that novel treatments may improve high Transcranial Doppler (TCD) flow velocity, leg ulcers and renal dysfunction in SCD patients (Runge et al., 2022); but, the options for stroke prevention in SCD are currently limited to hydroxycarbamide and blood transfusion, and additional research is needed to evaluate the role of aspirin and anticoagulation in SCD stroke prevention.

In brief, the benefits and risks of these treatments should be carefully evaluated in each individual case, considering the potential complications in long-term use. Plus, cost-effectiveness analyses have been conducted for various interventions in SCD, including blood transfusions, pharmaceuticals, and screening programs (Jiao et al., 2021). Future analyses should adopt a societal perspective, comprehensively model SCD complexity, and evaluate the impact of treatment on both quality of life and overall wellbeing. Although multiple new therapies for SCD have emerged and may become more widely used, these interventions may be limited and only exclusive to high-income countries. In order to make promising clinical trials of future curative treatments accessible to those in need, it is important to consider pharmacoepidemiology and pharmacoeconomic factors in SCD interventions.

## 7 Gene therapy as a therapeutic method

Gene therapy is a curative approach that employs genetic material to modify the progression of a medical condition. It involves introducing DNA or RNA into an individual’s cells. Gene therapy products are continually evolving and are primarily targeted towards cancer treatment. The introduction of genetic material can potentially affect not only the patient but also future offspring. Gene therapy can be performed in body cells or within egg or sperm cells. It possesses the capability to address the underlying origin of illness and enhance the wellbeing of individuals (Chen et al., 2023).

### 7.1 Different approaches used in gene therapy: gene addition

Gene therapy for SCD mainly involves two strategies: gene addition and gene editing. The principle of gene addition involves adding a functional copy of a gene to neutralize the disease phenotype and restore the normal function. This strategy is primarily used for recessive monogenic disorders resulting from non-functional mutant genes (Gonçalves and Paiva, 2017). The therapeutic gene that is delivered in monogenic recessive disorders is the normal wild-type form of the gene when the causative mutated gene is nonfunctional (Kirschner and Cathomen, 2020). Gene replacement therapy aims to deliver an accurate gene copy to restore the production of the faulty or absent protein and reverse the disease phenotype (Das et al., 2015). This approach has been successful in treating certain diseases such as hemoglobinopathies and immunodeficiencies (Kunz and Kulozik, 2020).

In addition, gene therapy utilizes various methods for gene addition. Both viral and non-viral vectors are incorporated in these techniques. Adenovirus, adeno associated virus, herpes simplex and retrovirus are frequently employed as viral vectors for gene delivery (Ghosh et al., 2020). Meanwhile, physical and chemical methods are further classifications for non-viral vectors (Attar, 2017). A few examples of physical techniques are laser beam, magnetofection, sonoporation, electroporation, particle bombardment gene guns and microinjection. In contrast, liposomes are used in chemical procedures in place of viral vectors. To enable gene transfer, these vectors need to have specific characteristics, such as masking DNA’s negative charge, condensing the molecule, and shielding it from enzymatic breakdown. Not just limited to the traditional viral vectors, researchers are also investigating the potential of synthetic transfection systems that incorporate physical, chemical, or electrical techniques. These alternative approaches offer potential safety benefits (Butt et al., 2022).

### 7.2 Evolution of gene therapy

Gene therapy has evolved significantly, progressing from gene transfer vectors for gene addition strategies to the use of genome editors for precise modifications. The discovery of molecular tools for genome alteration of hematopoietic stem cells and advances in genomic sequencing, which have deepened understanding of hemoglobin control, are driving the change in gene therapy procedures (Abraham and Tisdale, 2021). The latest gene editing techniques and next-generation lentivirus vectors have replaced the original usage of γ-retrovirus vectors. Insufficient transgenic expression, complicated cellular abnormalities, and difficulties in obtaining efficient and long-lasting suppression of hemoglobin S polymerization are some of the barriers that have led to the shift towards gene editing techniques (Ribeil et al., 2017). The use of gene editing techniques, such as CRISPR/Cas9 and Base Editors, shows promise in overcoming these challenges and improving the efficacy and safety of SCD gene therapy (Park and Bao, 2021).

The main reason for evolution of gene therapy as SCD treatment over time is to optimize its benefit-risk profile and improve outcomes. Initial patients in the HGB-206 study showed a modest expression of the anti-sickling hemoglobin (HbAT87Q), leading to alterations in the treatment process for subsequent patients (Kanter et al., 2023). These changes included improvements in cell collection and manufacturing, resulting in higher levels of HbAT87Q along with increased clinical and biologic effectiveness in Group B patients. The safety of the treatment largely reflected known side effects of the procedure, with no evidence of insertional oncogenesis. With the discovery of gene therapy for SCD, advancements in precision therapy, hematopoietic stem cell transplantation, and gene editing techniques have also been achieved (Abraham and Tisdale, 2021). These advances have expanded the potentially curative options for SCD patients.

Moreover, newer gene therapy approaches for SCD offer several advantages. One advantage is the use of lentiviral vectors to transduce autologous hematopoietic stem and progenitor cells (HSPCs) with anti-sickling β-globin genes, which has shown promising results in correcting the sickling phenotype and reducing HbS levels (Brusson et al., 2023; Ramadier et al., 2022). Combining gene addition and gene silencing strategies, like using βAS3-globin-expressing bifunctional lentiviral vectors and artificial microRNA (amiRNA) to suppress βS-globin production, confers additional benefits. This technique has successfully corrected the sickling phenotype and demonstrated a substantial decrease in HbS + red cells and βS-globin transcripts (Brusson et al., 2023; Finotti and Gambari, 2023). Furthermore, these newer gene therapy approaches have shown a standard integration profile, with no adverse effects of multilineage differentiation, engraftment, HSPCs viability, or the erythroid transcriptome and miRNAome, confirming their safety (Brusson et al., 2023). Therefore, new developments in gene therapy have the potential to enhance the effectiveness of existing SCD therapies without escalating the mutagenic vector burden.

### 7.3 Gene therapy as potential treatment in SCD

Gene therapy approaches for treating SCD include infusion of genetically modified autologous hematopoietic stem and progenitor cells (HSPCs) that express anti-sickling β-globin (βAS) after being altered using lentiviral vectors (LVs) (Ribeil et al., 2017; Germino-Watnick et al., 2022). LVs have shown success in inducing fetal hemoglobin production and have progressed to human trials. This strategy aims to reduce the levels of sickle hemoglobin (HbS) and promote the incorporation of functional βAS3-globin into Hb tetramers (Finotti and Gambari, 2023; Maruyama et al., 2015). Other strategies involve reactivating γ-globin expression and replacing the defective β-globin chain by gene editing with CRISPR-Cas nucleases or base editors (Park and Bao, 2021). The suppression of γ-globin expression can be accomplished by the disruption of cis-regulatory elements like LRF binding sites or BCL11A, or by reducing the expression of γ-globin in human erythroblasts through lineage-specific disruption of BCL11A (Park and Bao, 2021; Finotti and Gambari, 2023; Germino-Watnick et al., 2022; Cavazzana et al., 2017). These different types of gene therapy approaches have proven their effectiveness and safety in both preclinical and clinical studies; hence offering potential therapeutic opportunities for SCD treatment.

## 8 Approach of gene editing in propelling SCD treatment

A few decades back, recent applications have been actively used to develop direct editing in the cell genome. Several techniques for treating sickle SCD have used novel techniques for direct editing of cell genomes. Site-specific nucleases (SSNs) that are genetically created have the ability to direct editing, preferably to a single base pair throughout the whole genome. The transcription activator-like effector nucleases (TALENs), zinc finger nucleases (ZFNs), and CRISPR-Cas9 (clustered regularly interspaced short palindromic repeats-CRISPR-associated nuclease 9) are examples of these SSNs. The single-guide RNA (sgRNA), which will be present temporarily to initiate editing, is capable of being introduced into target hematopoietic stem and progenitor cells, electroporation of expression plasmids, *in vitro* transcribed mRNA, or implemented ribonucleoprotein complexes of recombinant Cas9 protein were used to generate HSPCs. However, the SSNs do not remain in the cells indefinitely.

The phrase “gene editing” is most commonly used in the context of sickle cell disease (SCD) to refer to a method of gene disturbance. Targeting HbF suppressors can enhance HbF while decreasing HbS. Targeting particular DNA segments inside a gene can be accomplished by combining an enzyme that cuts DNA and causes double-strand breakage with a guide that has great selectivity for identifying and firmly binding to the target. The sequence may be changed with high precision by creating a precise incision in the DNA, which frequently results in insertion and deletion. This kind of gene therapy frequently targets a unique area of DNA (aside from the HbS mutation) in order to enhance the creation of HbF while concurrently lowering the production of HbS. In particular, a lot of the treatments available now focus on the BCL11A gene, which is a HbF negative regulator. In this case, HbF production is increased by using gene editing to turn off HbF regulation.

## 9 Double strand break repair pathways

**FIGURE 2:**
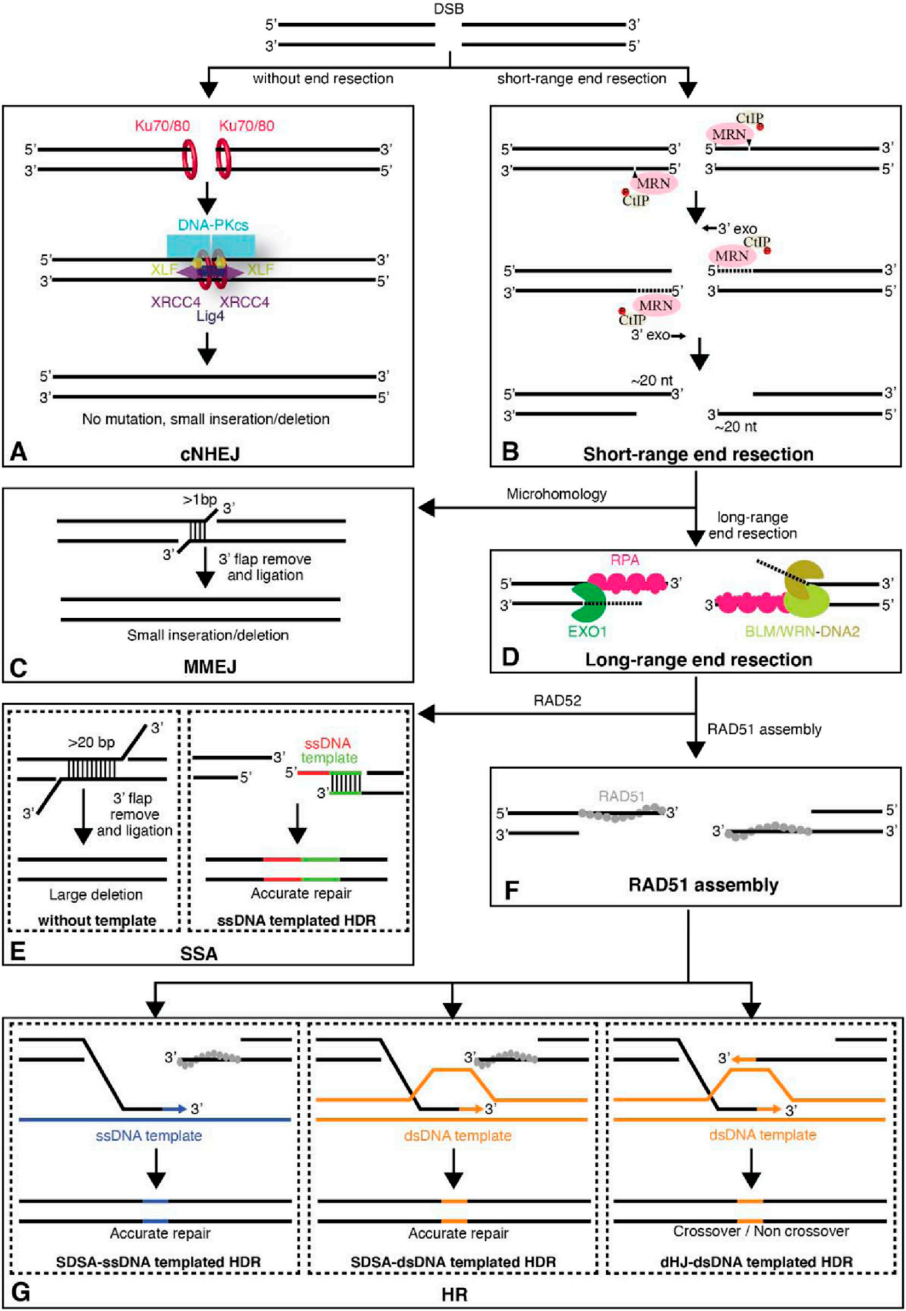
The major pathways of DNA DSBs (Double-Strand Breaks). **(A)** Unprocessed DSBs can be repaired through classic nonhomologous end joining (cNHEJ) allowing the two ends of the DSB to be re-ligated. **(B)** DSB ends can also be processed by the MRE11-RAD50-NBS1 (MRN) complex and its interacting factors to yield short 3′ single-strand (ss)DNA overhangs. **(C)** The short 3′ ssDNA overhangs can then be channeled into the microhomology-mediated end joining (MMEJ) pathway. **(D)** Alternatively, the DSB ends can undergo further long-range resection by either EXO1 or Bloom helicase (BLM)/DNA2. These longer ssDNA overhangs are first bound by replication protein A (RPA) and can then be channeled into the **(E)** single-strand annealing (SSA) pathway, which is mediated by the protein RAD52. **(F)** Alternatively, the RPA-ssDNA can serve as a substrate for RAD51 filament assembly, allowing the resulting DNA intermediates to be directed towards repair by **(G)** homology repair (HR). For HR, both ssDNA and dsDNA templated homology-directed repair (HDR) pathways are shown. Abbreviations: CtIP, C-terminal binding protein interacting protein; DNA-PK, DNA-dependent protein kinase; XLF, XRCC4-like factor; XRCC4, X-ray cross complementing group 4. Taken from Xue and Greene (2021) with permission.

The pathway explaining these conditions for gene editing includes non-homologous end joining (NHEJ), which causes certain bases (indels) to be inserted or deleted at the repair site by reannealing the ends of the DNA, frequently in an error-prone way. When multiples of three bases are introduced or removed the insertions may cause perturbation of the target gene, affecting the translational reading frame or adding or deleting certain amino acids in the translated protein. In contrast to that, according to a study in 2015, several organizations are working on ways to suppress NHEJ in order to promote the far more accurate repair that is accomplished by HR, as NHEJ is an erroneous repair mechanism (Maruyama et al., 2015). For example, LigIV activity may be blocked using small compounds to boost HR-mediated gene editing in mammalian cells, or LigIV can be stimulated to be broken down by proteases (Figure 2) (Xue and Greene, 2021).

Homology directed repair (HDR), which fills in the break gap by using a template from DNA supplied by a sister chromatid during cell division or given as an extra nucleotide sequencing reagent to function as a donor of the required sequence change, is another DNA repair mechanism that may be activated. Since DNA replication only takes place during the S and G2 phases of the cell cycle, only actively cycling cells can use HDR to modify genes. A double-strand break (DSB) close to an editing target location significantly boosts HDR’s efficacy.

**FIGURE 3:**
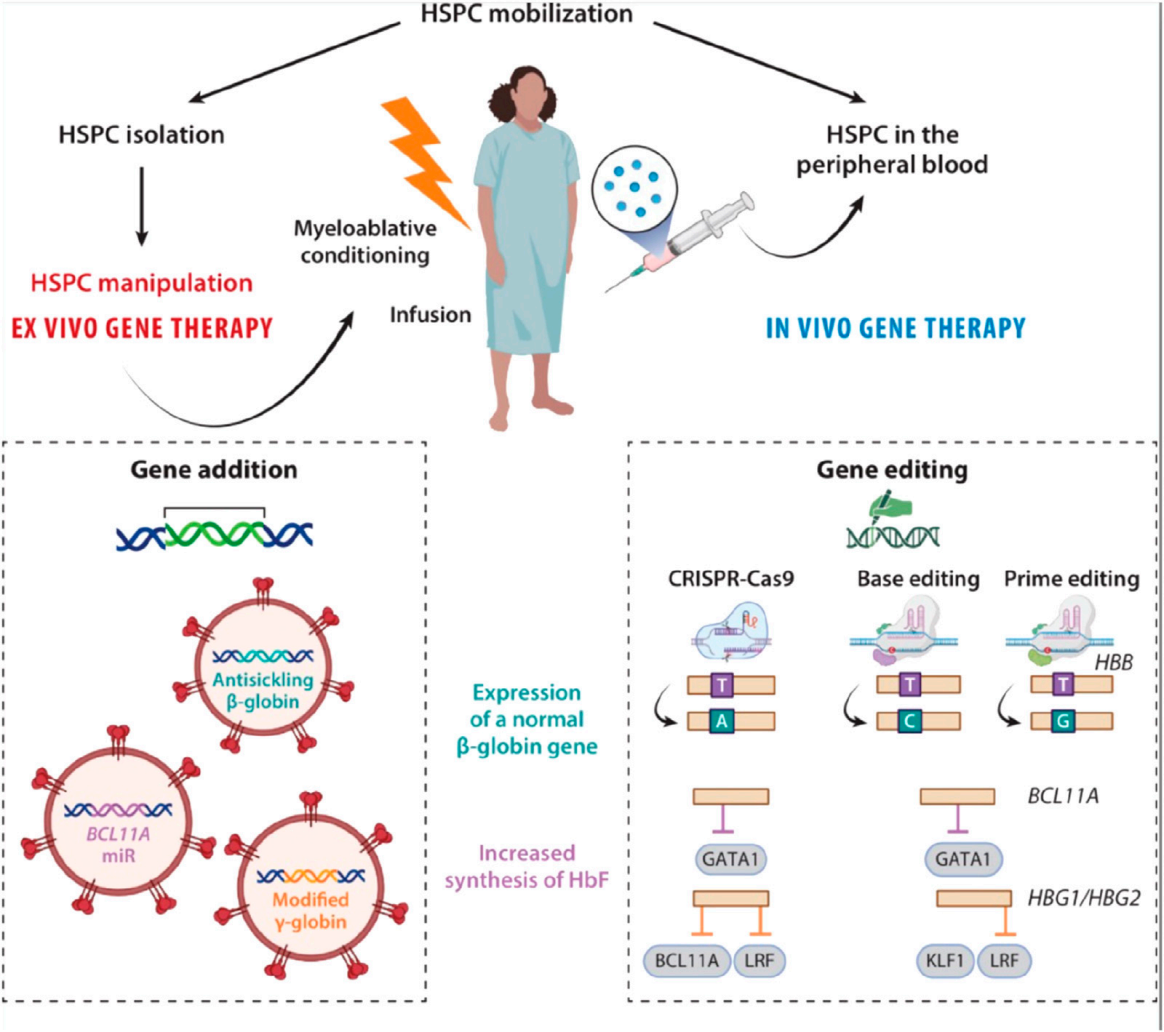
*Ex vivo* and *in vivo* approaches gene therapy for SCD. During *ex vivo* gene therapy, HSPCs are mobilized, isolated, and genetically modified outside the body. The patient undergoes myeloablative conditioning before being infused with the gene-corrected cells. *Ex vivo* gene therapy can be based on gene addition (with a lentivirus-based vector) or genome editing. *In vivo* gene therapy introduces the drug product directly into the HSPCs during their mobilization. It uses lentivirus-based gene therapy or genome editing and does not require conditioning. In the context of SCD, two strategies are used: expression of a normal β-globin gene or increase in HbF synthesis. Expression of a normal β-globin gene: In the gene addition approach, a lentiviral vector is used to deliver a β-globin antisickling transgene into the HSPCs. In a gene-editing approach, the SCD mutation in the β-globin gene can be corrected by HDR with a donor DNA template carrying the wild-type allele or by an adenine base editor (A > G), resulting in the production of the HbG-Makassar variant. Prime editing can precisely revert the SCD mutation (A > C). Increased synthesis of HbF: In a gene addition approach, the introduction of a modified γ-globin transgene or of a miR downregulating *BCL11A* (the HbF repressor) into the HSPCs induces the synthesis of HbF in their erythroid progeny. In a gene-editing approach, CRISPR-Cas9 nuclease indels are induced to disrupt the GATA1 activator binding site in *BCL11A*’s erythroid-specific enhancer. Another CRISPR-Cas9 nuclease-based strategy involves inducing indels in the *HBG1* and *HBG2* promoters to disrupt the HbF repressors’ (e.g., BCL11A or LRF) binding sites. Base editing generates *de novo* a DNA motif recognized by transcriptional activators (e.g., KLF1) or disrupts binding sites for transcriptional repressors (e.g., LRF) of HbF by inducing HPFH mutations in the *HBG* promoters. Abbreviations: Cas, CRISPR-associated protein 9; CRISPR, clustered regularly interspaced short palindromic repeat; HbF, fetal hemoglobin; HDR, homology directed repair HPFH, hereditary persistence of fetal hemoglobin; HSPC, hematopoietic stem and progenitor cell; indel, insertion and deletion event; LRF, leukemia/lymphoma-related factor; miR, microRNA; SCD, sickle cell disease. Taken from Cavazzana et al. (2025).

Additionally, there is evidence supporting base alteration as a more prominent method of modifying single base pairs. Engineers adopt a fusion protein that inculcates a combination of an enzyme that degrades a nucleotide (cytosine deaminase or adenosine deaminase) with Cas9, which locates the genomic target using sgRNA. For the purpose of generating a single-stranded nick rather than a double-strand break, one of Cas9’s nuclease domains is cut off. Instead, the base that has to be changed via deamination is “situated” adjacent to the enzyme deaminase (Hess et al., 2017). When the mutant Cas9 creates a single-stranded nick on the opposite strand, deaminated cytosine and deaminated adenosine are read as uracil and guanine, respectively. Base pair alterations (C:G to T:A or A:T to G:C) may be generated via this procedure. Aside from that, a number of architectural modifications to the Cas9-deaminase molecules have resulted in the production of highly active enzymes that predominantly work at the specified target site (Figure 3). Complicated sequence overwrites can be accomplished by prime editing and other base editing variants; sickle cell disease gene editing will most likely employ these techniques. There is further work to be done on further base editing technological developments.

The studied target that was mentioned above in SCD is the BCL11A gene which acts as a transcriptional factor that is used to suppress fetal (y-)globin expression, as previously mentioned. The erythroid enhancer of BCL11A is the object of clinical efforts employing ZFN and CRISPR-Cas9 to maintain its expression in non-erythroid blood cell lineages, where it is required for appropriate stem cell function and multi-lineage differentiation. The BCL may be disrupted very effectively by using HSPC manipulation approaches along with optimized CRISPR editing reagents. Results from one clinical research in 2021, using CRISPR-mediated disruption of BCL11A revealed that BCL11A erythroid enhancer is present in a significant proportion of treated HSPCs that maintain their ability to engraft and function as stem cells. It was shown that autologous HSPCs from two patients—one with severe ß-thalassemia and the other with sickle cell disease—had their erythroid enhancer of BCL114A disrupted *ex vivo* via CRISPR-Cas9 (Frangoul et al., 2021; Esrick et al., 2021). Those patients elucidate a dominant scale of fetal globin through their circulating erythrocytes thus, the SCD patients do not experience further veno-occlusive episodes (VOEs) which are episodic macrovascular occlusions.

Moreover, the majority of SCD patients may be cured with autologous transplantation of gene-edited hematopoietic stem cells, thanks to the development of CRISPR/Cas9 technology. But there are other obstacles to overcome before the gene-editing-based SCD therapy approach can be used in clinical settings, including as the requirement for high editing efficiency and minimal off-target consequences (Park and Bao, 2021).

## 10 Lentiviral vectors in gene editing

One promising treatment for sickle cell disease (SCD) is gene therapy, which involves the use of genetically modified cells (Cavazzana et al., 2025). This therapy involves introducing a novel gene into stem cells, typically by the use of a viral vector to transfer a non-sickling globin gene. Both the new hemoglobin and the native HbS are produced as a result of this process, which does not modify the native HbS gene. A lentiviral vector (LVV) is used in several current gene addition therapy projects to contain and transport a new gene. In short, this curative approach initiates the incorporation of globin genes to the genetically modified hematopoietic stem cells with viral vectors. Initially, this technology focused on y-retroviruses for clinical gene therapy but nowadays it has shifted to lentiviral vectors (LVs). Derived from retroviruses, this vector has shown promising clinical potential due to its ability to reliably deliver larger and complex DNA cassettes. For globin vectors to exhibit increasingly at a high level, this competency is essential.

Lentiviruses are also capable of transduction and integration into nondividing HSCs, where they support stable transgene expression files rather than retroviruses that fail due to their preference for specific gene misses and their inability to generate LVs at high titers in the absence of potent enhancer elements. Across areas of actively expressed genes with higher chromatin accessibility is typically the integration profile of LVs (Naldini et al., 2016). However, since LVs have the ability to cause insertional mutagenesis, safety concerns persist even with a better adaptation profile. LVs have been altered to become self-inactivating in order to address this. The deletion of the viral enhancer and promoter regions limits the long terminal repeat’s (LTR) ability to cis-act on cellular genes next to vector integration sites.

In contrast, LVs have the potential to cause insertional mutagenesis, safety issues are raised even with a safer adaptation background. LVs have been modified to become self-inactivating in order to counteract this. The deletion of the viral enhancer and promoter regions limits the long terminal repeat’s (LTR) ability to cis-act on cellular genes next to vector integration sites. In the vector plasmid, an additional change to the 5′LTR has also been made, with the CMV promoter taking the place of the U3 region. Through the changes that have been initiated, this will increase the safety profile and decrease the opportunity for the recombination to develop into replication-competent vectors. Research has been made, and the result exhibits hope for healing through autologous stem cell-based genome editing and additive gene therapy. Anti-sickling globin genes may be inserted into CD34 patient cells by lentiviral vectors. According to clinical investigations, there are fewer hemolysis and acute episodes when HbS is decreased to less than 50% (Steinberg, 2020). The most evidence has been gathered thus far from ongoing clinical studies using lentiviral gene therapy, which adds a modified β-globin gene (HbAT87Q) and has shown promise in reducing substantial VOEs in sickle cell disease. The conclusions of increases in organ function and long-term durability, however, are still preliminary (Kanter et al., 2023).

Other clinical trials also include four individuals with β-thalassemia who require transfusions have been administered LentiGlobin BB305 (Ribeil et al., 2017). These individuals no longer need frequent transfusions and have no clinically significant consequences. These results align with preliminary data from 18 more thalassemia patients who received LentiGlobin BB305 in clinical trial HGB-204.

Extensive research has been conducted on *ex vivo* gene therapy using globin gene insertion, and ongoing clinical studies are yielding positive results. Induced pluripotent stem cells and patient-derived hematopoietic stem and progenitor cells may now be able to undergo genetic correction thanks to the quick and significant advancements in genome engineering technologies, especially CRISPR/Cas9 (Demirci et al., 2018). But these methods are still in their early stages, and before implementing these promising methods in clinical settings, safety and effectiveness concerns need to be resolved.

## 11 Gene therapy in SCD

The knowledge that existing medicines do not make use of molecular or genetic expertise is disheartening, especially after 7 decades of knowing the hereditary basis of sickle cell disease (SCD). The human α- and β-globin gene clusters' shapes and DNA sequences were discovered more than 35 years ago, but their benefits for SCD patients are still unknown. There is new hope for people with sickle cell disease (SCD) and β-thalassemia thanks to recent advancements in gene therapy and the molecular genetics of hemoglobin expression (Salinas Cisneros and Thein, 2020).

Prior to the early application of gene therapy in sickle cell disease (SCD), the disease’s symptoms must first be treated. Supportive care is the mainstay of this approach, with an emphasis on pain management and penicillin prophylaxis for functional asplenia. Patients with recurrent splenic sequestration, intractable chronic pain, or stroke history should consider chronic transfusion. The mainstay of care for increasing fetal hemoglobin (HbF) levels is hydroxyurea (HU), an oral chemotherapeutic drug whose precise mechanism of action is yet unknown (Platt et al., 1984). Even though HU helps a lot of patients, it is not permanent treatment and using it does not deal with the underlying hematology of SCD.

While bone marrow transplantation is a therapeutic option, there are certain drawbacks, including the requirement for donor hematopoietic stem cells (HSCs) which are compatible with a body, the need for myeloablative preconditioning, and the possible problems such as graft-versus-host disease and loss of fertility. The difficulties are exacerbated by limited donor availability, especially in the groups most impacted by SCD. A viable treatment option that uses the patient’s own stem cells and eliminates the possible graft-versus-host disease is gene therapy.

Red blood cell lifespan and the characteristics of hemoglobin S (HbS) are related to all clinical symptoms of sickle cell disease (SCD). Interventions must either deliver non-sickling hemoglobin or prevent polymerization in order to reverse the underlying pathology. Nevertheless, the majority of the secondary or tertiary effects that are currently being treated, such as pain, inflammation, and leukocyte adhesiveness, point to the need for more thorough and focused care approaches for this complicated illness.

There are two primary types of efforts aimed at overcoming the polymerization property of hemoglobin S (HbS) in sickle cell disease (SCD). The first entails using structural information of the hemoglobin tetramer to develop small-molecule inhibitors of βS polymer formation. Some early initiatives, including the use of potassium cyanate, were not practical to provide for patients. With experiments on GBT440, a substance that modifies the oxygen-dissociation curve to inhibit deoxyhemoglobin polymerization, Global Blood, Inc. has recently brought this strategy back to life (Oksenberg et al., 2016).

The second group works on modifying red blood cell hemoglobin composition in order to modify the physiology of sickle cell disease. Through lentiviral transduction of CD34^+^ cells, traditional gene therapy introduces an extra globin gene (non-sickling β-chain or γ-chain for HbF) (Dong and Rivella, 2017). Utilizing gene editing to correct the βS mutation is an additional strategy. A different approach is to increase endogenous γ-globin gene expression by taking use of HbF’s strong antisickling properties (Hoban et al., 2016).

After then, the review focuses only on genetic strategies for bettering SCD treatment, with a particular emphasis on enhancing HbF expression. The discussion revolves around the progress made in comprehending HbF silencing during the fetal-to-adult transition, which has been fueled by genome-wide association studies (GWAS) (Manolio, 2010) and CRISPR/Cas9 (Doudna and Charpentier, 2014) gene editing technology. The regulation of HbF is discussed in detail in the next sections, where advancements in gene therapy and gene editing are highlighted as potential pathways toward better treatment for SCD patients.

### 11.1 Current update on gene therapy of sickle cell disease

In the past 20 years, gene therapy used for SCD commonly involved the lentiviral vector. The treatment has been found capable of curing SCD in both preclinical and clinical tests. The earliest SCD victim that was cured by lentiviral vector-mediated addition of an antisickling HBB into autologous HSCs had been observed to be a success. The results show that after 15 months, there is a great amount of therapeutic antisickling β-globin. Currently in clinical trials, LentiGlobin, has been assessed due to its safety and effectiveness. Nonetheless, the usage of lentiviral vectors contains possible risks like production of a replication-competent lentivirus (RCL) to infect unmarked cells, and insertional mutagenesis progressing towards genotoxicity. Even though brand new studies from clinical trials of lentiviral gene therapy show possibility of *ex vivo* engineering of autologous HSPCs, monitoring should be done to ensure resilience of the gene therapy with lentiviral-vector based (Park and Bao, 2021; Cavazzana et al., 2025).

As opposed to traditional gene therapy treatment, gene editing provides the possibility for irreversibly modifying the disease-causing genes by accurate addition, deletion, correction, and disruption of the target chain. Various gene editing approaches in SCD treatment have shown favorable results in preclinical studies. This include correction of contributing site of mutation in HBB, production of fetal hemoglobin (HbF) by gene-disruption of γ-globin (HBG) repressors, and by providing useful congenital endurance of fetal hemoglobin (HPFH) mutations located at locus of β-globin (Park and Bao, 2021).

#### 11.1.1 Ongoing clinical trials for gene therapy SCD

The earliest corporation to cure SCD patients by gene therapy is Bluebird Bio. The vector, LentiGlobin BB305,66 shows antisickling β-globin (T87Q). It reform locus control region (LCR) and β-globin promotor regulatory element without insulator sequence (Hoban et al., 2016). The approach in the earliest SCD patient who aged 13 years old causes 24% antisickling (exogenous) hemoglobin 4 and half months after autologous hematopoietic transplantation with transduced CD41 cells. No adverse effects were reported in this patient. There are also two other trials although no results have been gained yet (Hoban et al., 2016).

Based on research done before, ZFNs and TALENs are delivered for correction of SCD mutation accompanied by a DNA donor template. There are different organizations that evolved ZFNs and TALEN to focus on the suppressor of HbF transcription or the suppressor binding location in order to produce HbF. Agents like ZFN also have been used to target BCL11A locus in phase 1 and 2 in clinical trials (BIVV003, clinicaltrials.gov). CRISPR/Cas9 gene-editing was studied to correct sickle mutation in HBB, creating adequate HbF level to undo sickling through focusing on HbF transcriptional suppressor, and provide advantageous HPFH mutation. During phase 1, a dire SCD victim (CTX001, clinicaltrials.gov) was treated using analogue CD34^+^ HSPCs to induce HbF expression (Park and Bao, 2021). Table 1 shows the past and present overview of clinical trials focused on the development of gene therapy.

**TABLE 1:** Gene therapy clinical trials for patients with SCD: gene addition clinical trials. Adapted from Dimitrievska et al. (2024) and Cavazzana et al. (2025).

Gene therapy approach	Strategy	NCT ID	Sponsor	Intervention/Treatment	Phase	Location	Study status
Gene therapy	Antisickling gene transfer	NCT02151526/HGB-205	Bluebird bio	Drug: LentiGlobin BB305 Drug Product	I/II	France	Completed
Gene therapy		NCT02140554/HGB-206	Bluebird-bio	Genetic: lovo-cel	I/II	United States	Active, not recruiting
Gene therapy		NCT02247843	Donald B. Kohn, M.D., University of California, Los Angeles	Biological: βAS3-FB vector transduced peripheral blood CD34^+^ cells	I/II	United States	Active, recruiting
Gene therapy		NCT03964792	Assistance Publique–Hôpitaux de Paris	Genetic: DREPAGLOBE drug product	I/II	France	Completed
Gene therapy		NCT04293185/HGB-210	bluebird bio	Genetic: bb1111	III	United States	Active, recruiting
Gene therapy	Knockdown of *BCL11A*, γ-globin gene repressor	NCT03282656	David Williams, Boston Children’s Hospital, Los Angeles	shRNA targeting BCL11a - BCHBB694	I	United States	Active, not recruiting
Gene therapy	shRNA-based gene silencing	NCT05353647	David Williams	Biological: Autologous CD34^+^ HSC cells transduced with the lentiviral vector containing a shRNA targeting BCL11a	II	United States	Active, recruiting
Gene therapy	γ-globin gene transfer	NCT02186418	Children’s Hospital Medical Center, Cincinnati	Genetic: ARU-1801	I/II	United States	Completed
Gene therapy	γ-globin gene transfer and knockdown of *BCL11A*, γ-globin gene repressor	NCT04091737	CSL Behring	Human γ-globin G16D and shRNA734	I	United States	February 2019/May 2021 Terminated for unanticipated delays, not for safety reasons
Gene editing	Adenine Base Editor (ABE)	NCT05456880	Beam Therapeutics Inc.	Biological: BEAM-101	I/II	United States	Recruiting
		NCT03745287	Vertex Pharmaceuticals Incorporated	Biological: CTX001	II/III	United States	Ongoing

Gene-editing approach utilized engineered nucleases to create a DNA double-strand break (DSB) at an individual-specific site. This innovation allows irreversible mending of mutations that are contributed by SCD via editing of targeted sequence through DSB production continued with NHEJ or HDR. Definite DNA binding domains are present in ZFNs and TALENs and use FokI endonuclease domain. FokI is important for cleaving DNA. Although, the nucleases procedure is intricate, it needs a lot of time and proficiency. The class of nuclease that has been reviewed to be the most efficient currently is the CRISPR/Cas9 system (Park and Bao, 2021).

CRISPR/Cas9 uses single guide RNA sequences (gRNA). gRNA attaches at a particular marked location inside the gene and binds to Cas9 endonuclease. Next, Cas9 endonuclease will be directed towards a particular location through homology between gRNA and targeted DNA sequence. Even though there could be an issue from the action of off-target, the effect can be prominently decreased through rational gRNA composition or using high-accuracy of Cas9 protein. Base editors created from nucleotide deaminase with adjuvant paralyzed Cas9 protein can change bases directly with the lack of DSBs induction and HDR. This will enable the amendment of point mutation in indivisible cells. Thus, base editors are advantageous DNA editing equipment than Cas9 nuclease because Cas9 can cause production of undesired small insertions and deletions (indels), chromosomal rearrangements or translocations (Ramli, 2022).

#### 11.1.2 CRISPR/Cas9 system

CRISPR/Cas9 mechanism in gene editing can be considered the least intricate to produce gRNA. The significance of gRNA is to pilot the tracrRNA-crRNA chimera complex to a specific DNA strand in order to produce a targeted double-stranded DNA breaks (DSBs). The gRNA acts as a mark on the direction the complex should go and the Cas9 is the endonuclease responsible for breaking the DNA strand. Consequently, the cell will attempt to fix itself through Homology Directed repair (HDR) mechanism. There are two ways a gene can be adjusted in treatment of SCD: (i) CRISPR/Cas9 repair hemoglobin S into normal HbA and: (ii) induce HbF (Ramli, 2022).

The first approach involves repairing the HbS gene into normal β-globin protein. The approach required Cas9 protein, gRNA, and donor DNA simultaneously. Delivery of Cas9/gRNA into a cell as a ribonucleoprotein complex with donor DNA will enhance DSB rate and improve gene targeting frequency (Ramli, 2022). In the past years, there have been optimizations of CRISPR/Cas9 gene editing and delivery methods to enhance the efficacy of genome editing in HSPCs. In preclinical studies, there are many trials using plasmid DNA based systems for expression of Cas9 and gRNA, causing low gene editing efficiency and safety profile. Therefore, studies were done to enhance the overall quality of gene editing. Despite all editing techniques producing damage to the cell, transportation of Cas9 and gRNA as a pre-complexed ribonucleoprotein (RNP) can be considered acceptable in CD34^+^ HSPCs, although producing DNA damage response (DDR). A technique called as electroporation via nucleofection arrangement has been studied to be the favored procedure to deliver RNPs to HSPCs directly because RNP is able to invade the cell nucleus at a shorter time to immediately begin cutting the genome (Park and Bao, 2021).

The modification of mutation contributed by sickle cell utilizing editing of genes is known to be the most direct therapeutic pathway. CRISPR gRNA/Cas9 RNP complex targets the HBB jointly with donor template of DNA to be sent inside secluded HSPCs, causing HDR mediated correction of contributing mutation. There are various viral based vector choices tested for transport of donor template like adeno-associated viruses (AAVs). The main benefit of AAV is the small recurrence of vectors incorporated inside host genomic DNA. There is also a small likelihood of related insertional mutation of gene and genotoxicity. There is various research showing the effectiveness of marked incorporation at the locus of HBB in CD34^+^ HSPCs via RNP mixed with single-stranded oligodeoxynucleotides (ssODNs). Many studies used rAAV6 and ssODNs donors due to its safety profile and efficacy. Evolved erythroblasts from cells that undergo gene editing had a rise in HbA mean quantity and decreased phenotype of sickle cell. Those cells that undergo gene editing from SCD individuals are capable of engraftment in NSG or NBSGW mouse implant replicas. There is monitoring of the gene correction after the process of transplantation (Park and Bao, 2021).

The second approach involves the induction of HbF. Delivery of γ-globin gene is silenced and changed with β-globin gene is important to create HbA. Therefore, decreasing the factor expression that are responsible to silence γ-globin gene may contribute in creating more γ-globin gene, BCL11A is one of the factors that maintain HbF production in postnatal cell and does not give any side effects during erythropoiesis (Ramli, 2022).

#### 11.1.3 BCL11A gene disruption

Wu et al. (2019) showed very structured gene-editing in HSPCs by CRISPR/Cas9 disruption of GATA binding location at +58 BCL11A erythroid enhancer. This resulted in an erythroid specific decrease in expression of BCL11A. There is also an increase in production of the fetal γ-globin gene during engraving SCD HSCs. gRNA is responsible for cleaving the center of +58 erythroid enhancer of BCL11A directly. This resulted in the greatest level of HbF production in erythroid progenitors with a great amount of indels. Vertex Pharmaceuticals and CRISPR Therapeutics had produced favorable data in phase-1 (CTX001, clinicaltrials.gov) utilizing this approach (Park and Bao, 2021).

#### 11.1.4 Base editing of BCL11A

Base editing is capable of giving higher purity gene amended results than editing with nuclease. Base editors initiate switch in the base and avoid DSB producing unnecessary indels and off-targets. The A3A(N57Q)-BE3 base editor functions as RNP marking the BCL11A erythroid enhancer in SCD HSPCs. It aims for the cytosine to derange the GATA1. Electroporation within two rotations can increase the curative level. However, it can reduce the viability and potential of engraftment. Homozygous editing of one base at BCL11A enhancer in GATA1 motif may cause a strong HbF production which can be the same as nuclease editing (Park and Bao, 2021).

There is still a need for further investigation for base editing. Although off-target base editing can reduce subjection to RNP and use base editor jointly with weakened cytosine deaminase domain, detailed study is required before practicing in clinical procedure (Park and Bao, 2021).

#### 11.1.5 Induction HbF by introducing HPFH mutation

CRISPR/Cas9 causes disruption at the binding site of BCL11A binding site and at LRF in HBG promoters to increase induction of fetal hemoglobin. Research from Traxler et al. (2016) showed an innate 13 nucleotide (nt) HPFH in the HBG booster as the main aim. Following the editing via CRISPR/Cas9, HPFH elimination of 13-nt which is similar to naturally occurring mutation prevails amidst many indels. The location of Cas9 protein cleaves was surrounded with 8-nt tandem replicate which helped microhomology-mediated end joining (MMEJ) mending amended procreators synthesized blood cells for greater HbF amount which had been adequate to counteract sickling *in vitro.* Humbert et al. (2017) manipulates an NHP analogue implant replica for demonstrating the possibility of the procedure to treat SCD. Past evaluated target sites of CRISPR gRNA for humans had been utilized as the CRISPR target site. The target site is located at the promoter of analogous genes of HBG1 and HBG2. There has been observation of a reasonable number of large deletions because of simultaneous cleavage that causes removal of HBG2 gene as a whole and portion of the HBG1 promoter. Despite the common occurrence of huge deletions crucially decreased post-implant in NHP, the fundamental pathway remains unidentified. Besides, there is not enough data to determine the long term effect of this approach (Park and Bao, 2021).

## 12 Limitation and future potential treatment of SCD

Although gene therapy products lovotibeglogene autotemcel and exagamglogene autotemcel (Casgevy^®^) (a CRISPR/Cas9 genome-edited cell therapy) have been recently launched, however, the price is very high $3.1 million and $2.2 million, respectively (Vertex and CRISPR Therapeutics Announce US FDA Approval of CASGEVY™ … , n.d.) (Wilson and Little, 2025). Casgevy^®^ was recently marks it successful story in the treatment of SCD in Bahrain (Biospace, 2025). In contrast, gene therapy still requires a toxic conditioning regimen, and the long-term efficacy is not yet known (Curtis and Shah, 2020). Therefore, it is required to explore more affordable therapy for the diseases.

The future of treating sickle cell disease rests on cutting-edge approaches like gene therapy broadened hematopoietic stem cell transplantation strategies, and novel pharmacological agent. Although these innovations offer significant potential, it is crucial to tackle issues related to cost, accessibility, and long-term safety to ensure equitable benefits for all individuals affected by SCD.

## 13 Conclusion

To combat sickle cell disease two promising approaches have emerged: prenatal therapy and cord blood hematopoietic stem cells (HSC) treatments, particularly those employing CRISPR-Cas12 gene editing technology (Sanz Juste et al., 2023). Non-invasive prenatal diagnostics may allow for earlier and safer interventions, with CRISPR-Cas12 providing precise and efficient gene editing capabilities. However, this field demands careful ethical scrutiny, especially concerning informed consent and the potential long-term effects of fetal gene editing (Dimitrievska et al., 2024).

SCD presents significant clinical challenges due to its complex pathophysiology and varied clinical manifestations, advances in gene therapy hold the potential for transformative, curative treatments (Table 2). Ongoing research and clinical trials are critical to overcoming current limitations and achieving widespread clinical application (Orkin and Reilly, 2016; Dimitrievska et al., 2024).

**TABLE 2:** Summary of the aspect that being reviewed^a^.

Aspect	Details
Pathophysiology	HbS polymerization, vaso-occlusion, hemolysis, chronic inflammation
Acute complications	Acute chest syndrome, stroke, severe pain crises, infections
Chronic complications	Chronic anemia, nephropathy, pulmonary hypertension, bone necrosis
Gene therapy advances	CRISPR-Cas9, base editing, prime editing, lentiviral gene therapy
Challenges	Off-target effects, delivery efficiency, high costs

^a^ This table was generated from Scopus AI (www.scopus.com) based on keywords used.

In current SCD treatment, gene editing is more widely used compared with other methods while focusing on the class of nuclease that is considered to be the most efficient, which is the CRISPR/Cas9 system. The system utilized two approaches for treating SCD which focuses on the HbF production and correction of mutation into normal state cells. BCL11A is the key to increase production of HbF thus current clinical trials focus on BCL11A to show its therapeutic effects. Many studies approve the use of CRISPR/Cas9 for gene therapy.

Despite genetic knowledge, current treatments manage symptoms, but recent gene therapy offers hope using the patient’s own stem cells. Challenges with existing treatments and the promise of genetic strategies, particularly enhancing fetal hemoglobin expression, are highlighted. As clinical trials progress, questions about gene alteration risks and reimbursement issues emerge, emphasizing the need for effective and sustainable therapies. Expanding genetic therapy to areas with a high disease burden remains a key challenge.

SCD greatly impacts the quality of life of patients and their family. This occurred due to the pain and complications endured daily, as well as the social discrimination. Furthermore, the degree of severity of the disease and general health of the patient vary so they have different abilities to cope with the condition. Nevertheless, better approaches are still under observation to provide a higher safety profile and efficacy for the patients.
